# Graphical User Interface Development for a Hospital-Based Predictive Risk Tool: Protocol for a Co-Design Study

**DOI:** 10.2196/47717

**Published:** 2023-08-31

**Authors:** Nicholas Marlow, Marion Eckert, Greg Sharplin, Ian Gwilt, Kristin Carson-Chahhoud

**Affiliations:** 1 University of South Australia Adelaide Australia

**Keywords:** co-design, health information technology, health professional, user-centered design, user interface

## Abstract

**Background:**

This co-design research method details the iterative process developed to identify health professional recommendations for the graphical user interface (GUI) of an artificial intelligence (AI)–enabled risk prediction tool. Driving the decision to include a co-design process is the belief that choices regarding the aesthetic and functionality of an intervention are best made by its intended users and that engaging these users in its design will promote the tool’s adoption and use.

**Objective:**

The aim of this research is to identify health professional design and uptake recommendations for the GUI of an AI-enabled predictive risk tool.

**Methods:**

We will hold 3 research phases, each consisting of 2 workshops with health professionals, between mid-2023 and mid-2024. A total of 6 health professionals will be sought per workshop, resulting in a total enrollment of 36 health professionals at the conclusion of the research. A total of 7 workshop activities have been scheduled across the 3 workshops; these include context of use, notifiers, format, AI survey–Likert, prototype, AI survey–written, and testing. The first 6 of these activities will be repeated in each workshop to enable the iterative development and refinement of GUI. The last activity (testing) will be performed in the final workshop to examine health professionals’ thoughts on the final GUI iteration. Qualitative and quantitative results data will be produced from tasks in each research activity. Qualitative data will be examined through inductive thematic analysis or deductive thematic analysis in accordance with the Nonadoption, Abandonment, and Challenges to the Scale-up, Spread, and Sustainability (NASSS) framework; visual data will be examined in accordance with “framework of interactivity;” and quantitative data will be examined using descriptive statistics.

**Results:**

Project registration with the Australia and New Zealand Clinical Trial Registry has been requested (#384098). Finalized design recommendations are expected in early to mid-2024, with a results manuscript to be submitted in mid-2024. This research method has human research ethics approval from the South Australian Department of Health and Wellbeing (#2022/HRE00131) as well as from the Human Research Ethics Committee of the University of South Australia (application ID#204143).

**Conclusions:**

Understanding whether an intervention is needed in a particular situation is just the start; designing an intervention so that it is used within that situation is paramount. This co-design process engages end users to create a GUI that includes the aesthetic and functional details they need in a manner that aligns with their existing work practices. Indeed, interventions that fail to do this may be disliked, and at worst, they may be dangerous.

**International Registered Report Identifier (IRRID):**

PRR1-10.2196/47717

## Introduction

Dashboards summarize information in a meaningful manner to ensure users can make informed decisions in accordance with organizational objectives [1]. They can take many forms, such as electronic medical records (EMRs) or electronic health records (EHRs), as well as computerized provider order entry (CPOE), to name but a few. Each is advocated as a tool that increases productivity and efficiency, improves patient health care outcomes [2], and assists health professionals to avoid errors and drive down health care costs [3]. Yet despite their advocated benefits, health professionals encounter barriers to their use and sustainability [4].

In many instances, barriers result from dashboards’ imposition on the way health professionals deliver care or the way in which they are required to interact with the platform in their professional practice. A thematic analysis of physician interviews identified that using the platform not only disrupted their patient consultation but also resulted in an additional time burden due to the need to record diagnostic codes [5]. Confidence in the quality of information within these records is of concern for health professionals, with “prescriber” views reporting that patient files included “untrustworthy” information and produced “irrelevant” alerts [6]. Distrust as to the true intent of health information technology (HIT) has also been identified, with some health professionals reporting feeling that their CPOE systems are a mechanism for oversight and performance monitoring [7]. How health professionals interact with dashboards can also create issues, with research identifying pharmacist views that CPOE use can be time consuming [8] and a struggle due to functional and navigational issues [9].

Workplace practice and navigational issues, among others, can accumulate and result in a negative experience for health professionals. Of the interviewed physicians, 64.2% reported “agreeing” or “strongly agreeing” that EMRs added to the “frustration of their day.” Furthermore, these physicians also had significantly higher odds of burnout [10].

The introduction of artificial intelligence (AI)–enabled technologies in health care has been reported to raise several concerns for health professionals, specifically ethicality, data management, and its influence on health care delivery [11]. Alternatively, research on AI adoption reports that as health professionals’ exposure to AI increases, their positive feelings toward it also increase, as do their satisfaction and use intentions [12]. It is critical, therefore, that health professionals’ engagement with AI occurs in a positive manner to encourage adoption and repeated use.

Positive interaction with AI can be achieved through end-user engagement in graphical user interface (GUI) design. Indeed, the GUI is the most prominent, and perhaps the only, element of AI-enabled software with which the user will interact. Its appropriate design is therefore paramount. Co-design as a participatory process enables meaningful engagement with relevant users [13]. It is a process where the contributions of different users are synthesized [14], and it collaboratively integrates design thinking, stakeholder knowledge, scientific evidence, and the bottom-up approach of participatory action research to produce solutions to identify and address what is important within the local context [4]. Co-design will be used by this research to develop the notifiers (eg, graphs, dials, and icons) and layouts required because it places the user at the center of the research and development process with the goal of creating the version of the GUI that best represents their design needs.

The research method detailed herein will be performed as part of a PhD project and forms part of the larger Predictive Harm Response Management (PreHaRM) Algorithmic Tool to Reduce Adverse Events in Health Care Settings project. The purpose of the larger project is to develop and test an AI-enabled predictive risk algorithm and interface for use within the hospital setting (the PreHaRM tool). The PreHaRM tool will focus on the prediction of 3 adverse events: patient falls, medication errors, and occupational violence (patient or caregiver toward staff). The larger project, including this co-design project, is supported by Digital Health Cooperative Research Centre (DHCRC) Limited. DHCRC Limited is funded under the Australian Commonwealth Government’s Cooperative Research Centers Program (Project #DHCRC-0156). The co-design workshops will develop health professionals informed recommendations for the visualization of the DHCRC PreHaRM tool.

## Methods

### Objective

The aim of this research is to identify health professional design and uptake recommendations for the GUI of an AI-enabled predictive risk tool. The primary and secondary objectives are described in the following sections.

#### Primary Objective

Objective 1: Identify health professionals design preferences for GUI aesthetics and function.

#### Secondary Objectives

Objective 2: Identify health professional usability levels for professional practice and PreHaRM GUIs.

Objective 3: Identify health professional acceptability levels for professional practice and PreHaRM GUIs.

Objective 4: Identify health professionals’ perceptions of AI in health care in relation to its implementation and adoption.

#### Outcome Measures

Outcome measures developed in accordance with objective 1 are as follows:

- Participant for GUI aesthetic and function preferences tabulated and narratively synthesized according to the framework of interactivity.
- Participant visual design preferences tabulated and narratively synthesized according to the elements and principles of visual design.

Outcome measures developed in accordance with objective 2 are as follows:

- Participant System Usability Scale (SUS) scores for professional and PreHaRM GUIs.

Outcome measures developed in accordance with objective 3 are as follows:

- Barriers and enablers for professional and PreHaRM GUIs tabulated and narratively synthesized according to the theoretical framework of acceptability.

Outcome measures developed in accordance with objective 4 are as follows:

- Participant “AI potential impact” scores measured by the 10-question Shinners Artificial Intelligence Perception tool.
- Participant “AI readiness” scores measured using the 10-question Shinners Artificial Intelligence Perception tool.
- Barriers and enablers to AI adoption in health care tabulated and narratively synthesized according to the Nonadoption, Abandonment, and Challenges to the Scale-up, Spread, and Sustainability (NASSS) framework.

### Study Design

A total of three 2-month research phases have been scheduled from mid-2023 to mid-2024, with 2 co-design workshops scheduled per phase, one in Adelaide (University of South Australia city west campus), the other in Tonsley (Tonsley Innovation District) for Central Adelaide and Southern Adelaide Local Health Network staff, respectively. If required, participants may attend using teleconferencing software (eg, Zoom) and collaborative software (eg, Miro). Each workshop will be led by an experienced facilitator and run for approximately 2 and a half hours. A total of 2 support staff will also be in attendance: one will observe and note proceedings while a second will provide operational support to the facilitator (eg, information technology troubleshooting, handing out and collecting activity sheets).

Members of the PreHaRM clinical implementation team (CIT) will be asked to promote each workshop by circulating an invitation to colleagues within their peer network. Individuals who confirm their interest in the workshop will be emailed workshop details (ie, session date, time, and location). A total of 6 participants will be enrolled in each workshop, resulting in a total of 12 participants per research phase, for a maximum total of 36 participants (Table 1). The total number of individuals may be less than 36, as participants will be allowed to attend more than one workshop should they choose to; however, this repeat attendance is not a requirement and will be made at their discretion.

**Table 1:** Research phase and co-design workshop schedule.

Phase and location			Participants, n	Date^a^
**Research phase 1**				
	CALHN^b^ workshop 1	6		September
	SALHN^c^ workshop 1	6		September
**Research phase 2**				
	CALHN workshop 2	6		November or December
	SALHN workshop 2	6		November or December
**Research phase 3**				
	CALHN workshop 3	6		January or February
	SALHN workshop 3	6		January or February

^a^Proposed 2023 time period.

^b^CALHN: Central Adelaide Local Health Network.

^c^SALHN: Southern Adelaide Local Health Network.

### Inclusion and Exclusion Criteria

Nurses have been identified as the target user of the GUI, and as such, the inclusion and exclusion criteria below reflect the need to attract a diverse range of participants from this professional group; for example, nurses of all levels (including enrolled, registered, and nursing unit managers) will be sought so the diversity of their experience can benefit the design. Criteria for enrollment are listed in Textbox 1 below.

**Inclusion and exclusion criteria.**

**Inclusion criteria**

- Enrolled nurses
- Registered nurses
- Nursing unit managers
- Nurses in noncare roles
- Use health information technology in professional role
- Able to provide informed consent

**Exclusion criteria**

- Not employed by SA Health
- Unable to provide informed consent

### Iterative Workshops

**Figure 1:**
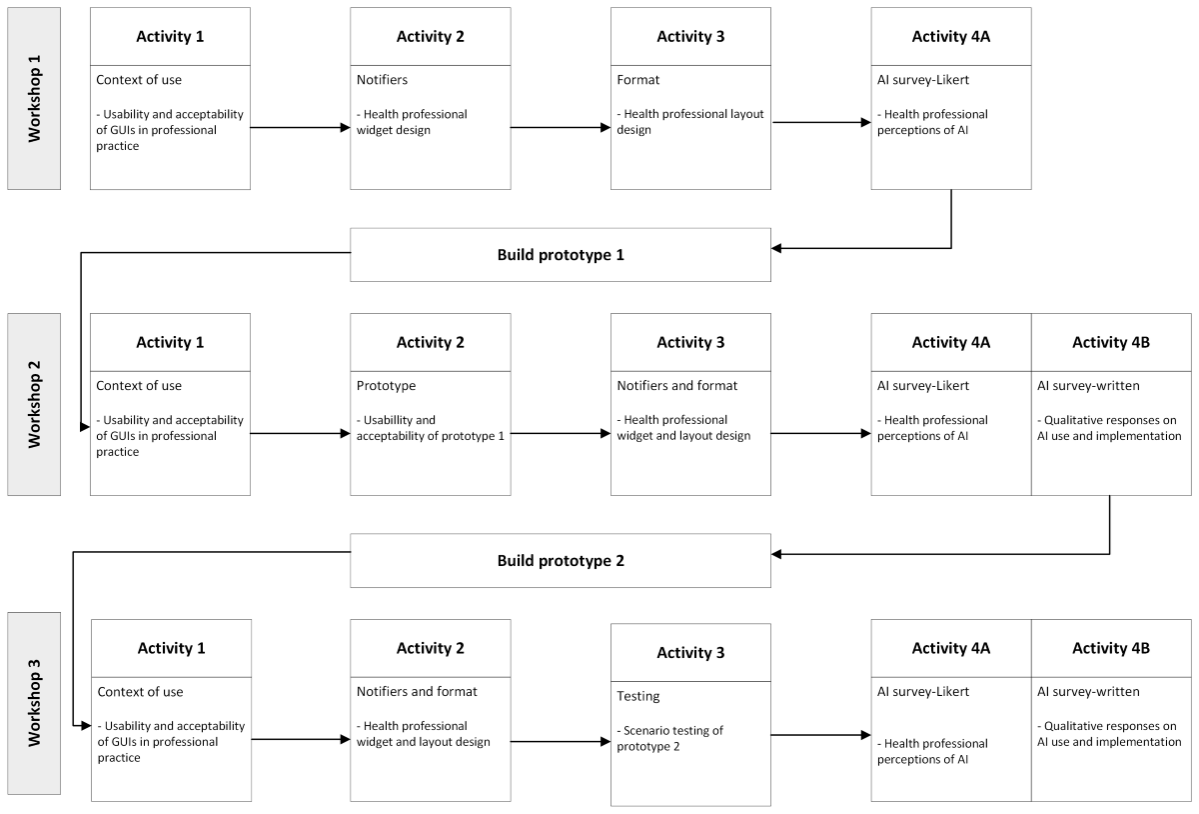
Iterative co-design process. AI: artificial intelligence; GUI: graphical user interface.

An iterative co-design process will run across each of the workshops. Each workshop includes predesign and generative activities, noting that these activities will culminate in the testing activity held in the third workshop. Figure 1 illustrates the focus and relationship of each workshop, as well as the overall iterative co-design process.

A description of each workshop activity is provided in Table 2 below.

**Table 2:** Workshop activity matrix.

Activity	Description	Tools
Context of use	Participants will be asked about their use of GUIs^a^ at work.	Web-based survey Moderator guide
Notifiers	Participants will be asked to design notifiers (eg, graphs, dials, or icons) that they would like to see on the PreHaRM^b^ GUI.	Generative toolkits
Format	Participants will be asked to arrange their chosen visualizations on a mock GUI.	Generative toolkits
AI survey–Likert	Likert scale questions will investigate participant feelings toward AI^c^ enabled software in health care.	Web-based survey
Prototype	Participants will be asked to provide feedback in relation to the potential usability of the PreHaRM prototype 1.	Web-based survey Moderator guide
AI survey–written	Participants will be given results from previously completed surveys and asked to contextualize the results from their point of view.	Web-based survey
Testing	Participants will be asked provide feedback on their use of prototype 2 in relation to a mock workplace scenario.	Scenario testing Web-based survey

^a^GUI: graphical user interface.

^b^PreHaRM: Predictive Harm Response Management.

^c^AI: artificial intelligence.

### Workshop Tools

Workshop tools and the method for analyzing their respective results data are described in the follows sections.

#### Surveys

A background survey will be used to obtain basic participant demographic information (workshops 1-3). The SUS will be used to examine participants views on the usability and acceptability of GUIs currently used in their professional practice (“context” activity, workshops 1-3) [15]; a sample survey has been included for reference (Multimedia Appendix 1). The SUS activity survey will also be used to understand participant views on the PreHaRM prototypes 1 and 2 (“prototype” activity, workshop 2, and “testing” activity, workshop 3). The Shinners Artificial Intelligence Perception (SHAIP) framework will be used to identify health professionals’ perceptions of AI in health [11] (AI survey–Likert, workshops 1-3). The third survey will present results from the AI survey–Likert alongside written questions asking participants to interpret and report what these results mean (AI survey–written, workshops 2-3). All surveys will be delivered using a web-based platform (eg, Microsoft Forms). All data will be downloaded from the web-based survey platform for analysis. Quantitative data collected from the background, SUS, and SHAIP surveys will be examined using descriptive statistics calculated in R (version 4.2.0; R Core Team). Qualitative data collected from the AI Survey–written will be coded against the 22 themes within the NASSS framework in NVivo (version 14; QSR International) [16]. Coding will be performed by NM with checking by a second researcher; any disagreements will be reviewed by KCC.

#### Moderator Guide

Health professionals’ acceptance of professional practice GUIs as well as the PreHaRM prototype GUI will be examined through questions contained in a semistructured moderator guide. Their responses will be audio recorded and transcribed, and the coding of responses will then be performed against the 8 domains of the theoretical framework of analysis (TFA) [17]. Coding will be performed by NM and checked by a second researcher, with any disagreements reviewed by KCC.

#### Generative Toolkits

For notifier development, participants will be given example cut-outs and asked to amend them to represent how they’d prefer it to look. Blank paper, colored markers, and other materials will be provided for participants should they prefer to draw their own notifiers. For layout development, participants will be given GUI screen templates (and blank pieces of paper) onto which they can arrange their chosen notifiers to design their preferred GUI (“notifiers” and “format” activities, workshops 1-3).

Notifiers and layouts will be produced using generative toolkits. Both artifact types will be assessed to identify their aesthetic and functional characteristics. Aesthetic characteristics will be examined in relation to the visual elements that comprise each artifact (ie, line, shape, color palette, texture, typography, and form) as well as the use of visual design principles (ie, unity, gestalt, space, hierarchy, balance, contrast, scale, dominance, and similarity) [18]. Functional (or interactive) characteristics will be examined using the framework of interactivity [19]; this will be used to categorize each artifact in regard to its ideational, interpersonal, and textual intent. All artifacts will be examined by NM, with results recorded on an Excel spreadsheet developed a priori. These will be checked by a second researcher, with KCC adjudicating any disagreements. Review of the content and frequency of these results will inform the PreHaRM design recommendations.

#### Scenario Testing

A mock scenario to test PreHaRM prototype 2 will be developed using CIT, the technical advisory group (TAG), and research staff input. Participants in workshop 3 will be offered 1 opportunity to attempt the tasks within the scenario (“testing” activity, workshop 3). All attempts will be video recorded, either through screen capture software or a separate video camera. Participant attempts will be examined using “navigation testing,” where participants’ time taken as well as navigation hits and misses will be recorded. Exploration results will be examined using descriptive statistics calculated in R. Following their completion of the scenario, they will be asked to complete the SUS questionnaire, and the resultant data will be examined as per the process described above.

### Unstructured Interviews

Following the completion of each workshop, attendees will be offered the opportunity to participate in voluntary, one-on-one, unstructured interviews with NM. The purpose of the interview is to enable participants to provide further information about their workshop feedback or to provide new feedback if they feel uncomfortable raising it in a group context. Interviews will be offered through teleconferences only (Zoom; version 5.12.8; Zoom Video Communications Inc). These interviews will be offered as an opt-in opportunity, and it will be explained that participation in any workshop is not contingent on their participation in the interviews.

In unstructured interviews, it is unclear what participants may choose to discuss; consequently, inductive thematic analysis will be used to examine participant interview transcripts. This will be performed using NVivo. Participant perspectives will be coded by NM and examined by KCC.

### Data Management

All workshops and follow-up interviews will be audio recorded using a digital recording device; scenario testing activities will be recorded using video cameras; hardcopy artifacts (ie, visualizations and dashboards) will be collected by PreHaRM researchers; and participant surveys will be collated using web-based software. Audio recordings will be transcribed using voice recognition Rev (Rev.com, Inc); video recordings will be reviewed using Adobe Premiere Pro (version 23.0.0; Adobe Inc); Microsoft Forms will host each survey and automatically collate responses. Once the survey period has ended, collated responses will be downloaded. All electronic data will be stored in password-protected folders at the University of South Australia, Australia. All artifacts will be stored in facilities at the University of South Australia, Australia. All data will be retained for a minimum of five years after project completion in accordance with Australian State and Commonwealth guidelines.

Peer-reviewed manuscripts, posters, and oral presentations detailing the co-design process and results will be produced; information used in these research outputs will be deidentified. If video recordings are used, then participant likenesses (audio and visual) will be obscured. Data sets generated and analyzed during this research are not publicly available due to dissemination restrictions resulting from the ethics approval process.

### Ethical Considerations

A participant information sheet and consent form will be emailed to all participants before each workshop. Hardcopies will be available at the start of each session, and all individuals must read the information sheet and sign the consent form before participating. This research method has human research ethics approval from the South Australian Department of Health and Wellbeing (#2022/HRE00131) as well as from the Human Research Ethics Committee of the University of South Australia (application ID#204143). No research activity will commence until approvals from both committees have been received. All research data will be deidentified by researchers. Participants will be offered an AU $50 (US $32.82) gift card for their attendance.

## Results

Recruitment for this project will commence in mid-2023, with data collection scheduled to commence in mid- to late 2023 and end in mid-2024. Co-design and review will occur throughout the data collection period.

## Discussion

### Strengths

The key strength of this research and the broader PreHaRM project is the purposeful engagement of end users in each research activity; this is research with health professionals for health professionals. Preliminary engagement with the state government and local health networks has been achieved through engagement with the CIT. Comprised of senior hospital staff and government health care data professionals (managers and stewards), this group provides strategic information on data collection and use within the local health care system. This insight provides PreHaRM researchers with a deeper understanding of the administrative and technical context in which health professionals work. Similarly, the PreHaRM TAG includes leaders in AI software development and interface design. This group guides researchers’ thinking on the capacity of the PreHaRM algorithm. Both the CIT and the TAG have already been central to the predesign phase [20] and will continue to remain involved in the upcoming stages of the design process. Health professionals drive the generative phase of the PreHaRM tool primarily through their efforts in the “notifiers” and “format” activities, but also through their descriptions of software use and their feelings toward AI within health care. The evaluative activities of researchers and the presentation of these results back to health professionals further involve them in the iterative and generative process at the crux of this research. The broad contextualization of the health care system and guidance of AI development from the CIT and TAG, respectively, are operationalized through the generative activities of health professionals at the “coal face.”

### Limitations

As primary end users, nurses are the target cohort for each workshop, and their input in these sessions will inform the PreHaRM GUI. Within the hospital context, however, it is possible that other health professionals may also use the PreHaRM GUI. Callen et al [21] identified that differences in organizational culture along professional lines represent differences in attitudes toward HITs. Therefore, the focused recruitment of one health professional group is a potential limitation; future research should broaden recruitment to capture the design requirements of additional health professional cultures. Additional in situ research should be conducted with health professionals to confirm the issues they noted in the sessions are as consequential as they are real and to seek further refinements that improve the adoption of the PreHaRM tool.

### Practical Significance

This project will engage health professionals in an iterative co-design process across 3 workshops. This process of repeat engagement will enable researchers to refine recommendations for the aesthetics and functionalities of the PreHaRM GUI. It is anticipated that by adopting their recommendations, health professionals will be more likely to accept and use this tool and to prevent forecast events from occurring. The target audience of this protocol is other health researchers, health professionals interested in research, and user interface and user experience design researchers.

### Conclusions

The PreHaRM tool will combine an AI-enabled predictive risk algorithm with a co-designed GUI. The purpose of this algorithm is to identify the potential occurrence of adverse events, whereas the purpose of the GUI is to convey these potential events to health professionals in a meaningful manner. Indeed, the presentation of the algorithm results is paramount if the warning it displays is to be acknowledged and actioned. Co-design will be used in this research to identify end user aesthetic and functionality design recommendations to help ensure the appropriateness of the final product and its incorporation with existing work practices. This research takes as a basic assumption that the PreHaRM GUI, designed with input from health professionals, will be used by professionals to the benefit of patients and their families.

## Appendix

Multimedia Appendix 1 Professional practice dashboard use web-based survey.

## Abbreviations

AI: artificial intelligence
CALHN: Central Adelaide Local Health Network
CIT: clinical implementation team
CPOE: computerized provider order entry
DHCRC: Digital Health Cooperative Research Centre
EHR: electronic health record
EMR: electronic medical record
GUI: graphical user interface
HIT: health information technology
NASSS: Nonadoption, Abandonment and Challenges to the Scale-up, Spread and Sustainability
PreHaRM: Predictive Harm Response Management
SALHN: Southern Adelaide Local Health Network
SHAIP: Shinners Artificial Intelligence Perception
SUS: System Usability Scale
TAG: technical advisory group
TFA: theoretical framework of analysis
