# Supplementary material for: Graphical User Interface Development for a Hospital-Based Predictive Risk Tool: Protocol for a Co-Design Study
Source: JMIR Res Protoc. 2023 Aug 31;12:e47717. doi: 10.2196/47717 (PMC10502603; doi:10.2196/47717)
Supplement: Multimedia Appendix 1 [file resprot_v12i1e47717_app1.pdf]

PreHaRM: Professional practice dashboard use

The following 10 questions come from the 'System Usability Scale' tool and are designed to identify your views on usability.

Please note that the information you provide will only be used in aggregate, and no information that will enable your identification will be published or disseminated to any party.

1. Please select a response from the following options that best represents your opinion on the dashboard you most frequently use in your work role.

	Strongly agree	Agree	Neither agree nor disagree	Disagree	Strongly disagree
I think that I would like to use this system frequently	○	○	○	○	○
I found the system unnecessarily complex.	○	○	○	○	○
I thought the system was easy to use	○	○	○	○	○
I think that I would need the support of a technical person to be able to use this system.	○	○	○	○	○
I found the various functions in this system were well integrated.	○	○	○	○	○
I thought there was too much inconsistency in this system.	○	○	○	○	○
I would imagine that most people would learn to use this system very quickly.	○	○	○	○	○
I found the system very [illegible]	○	○	○	○	○

cumbersome to use.	○	○	○	○	○
I felt very confident using the system.	○	○	○	○	○
I needed to learn a lot of things before I could get going with this system.	○	○	○	○	○
